# Radiolabeled Apoptosis Imaging Agents for Early Detection of Response to Therapy

**DOI:** 10.1155/2014/732603

**Published:** 2014-10-14

**Authors:** Kazuma Ogawa, Miho Aoki

**Affiliations:** Division of Pharmaceutical Sciences, Graduate School of Medical Sciences, Kanazawa University, Kakuma-machi, Kanazawa 920-1192, Japan

## Abstract

Since apoptosis plays an important role in maintaining homeostasis and is associated with responses to therapy, molecular imaging of apoptotic cells could be useful for early detection of therapeutic effects, particularly in oncology. Radiolabeled annexin V compounds are the hallmark in apoptosis imaging *in vivo*. These compounds are reviewed from the genesis of apoptosis (cell death) imaging agents up to recent years. They have some disadvantages, including slow clearance and immunogenicity, because they are protein-based imaging agents. For this reason, several studies have been conducted in recent years to develop low molecule apoptosis imaging agents. In this review, radiolabeled phosphatidylserine targeted peptides, radiolabeled bis(zinc(II)-dipicolylamine) complex, radiolabeled 5-fluoropentyl-2-methyl-malonic acid (ML-10), caspase-3 activity imaging agents, radiolabeled duramycin, and radiolabeled phosphonium cation are reviewed as promising low-molecular-weight apoptosis imaging agents.

## 1. Introduction

Apoptosis is known to play an important role in maintaining homeostasis; therefore, it is associated with several diseases and responses to therapy. Molecular imaging of apoptotic cells could be useful for elucidation of the diseases and early detection of therapeutic effects and could contribute to personalized medicine. In this review, we introduce radiolabeled compounds for molecular imaging of apoptosis (cell death), their applications, and utility in medicine.

## 2. Radiolabeled Annexin V

The lipid composition of the outer and inner leaflets of the plasma membrane is not symmetrical. Phosphatidylserine (PS) is normally retained on the intracellular face of the cell membrane. When cells undergo apoptosis, this distribution is altered, so that PS is rapidly exposed to the outside of the cell membrane. In fact, not only apoptosis but also some other forms of cell death including autophagy can externalize PS [1, 2]. Moreover, in the final stage of almost all forms of cell death, PS on the intracellular face of the plasma membrane become accessible to PS-targeting probes in a nonspecific fashion, because of complete loss of membrane integrity. Thus, PS-targeted radiolabeled probes must not be considered specific apoptosis imaging agents, but they may be used to detect multiple forms of cell death.

In this paper, we have used the phase “apoptosis imaging” for PS-targeted radiolabeled probes. However, to be accurate, it is “cell death imaging” and not “apoptosis imaging.” The typical compound of PS targeted carriers is annexin V, a 36 kDa human protein with a nanomolar affinity for membrane-bound PS [3–5]. Because of this property, radiolabeled annexin V compounds have been developed and evaluated as* in vivo* imaging agents for detection of cell death.

## 3. ^99m^Tc-4,5-bis(thioacetamido)pentanoyl-Annexin V (^99m^Tc-BTAP-Annexin V)

**Figure 1:**
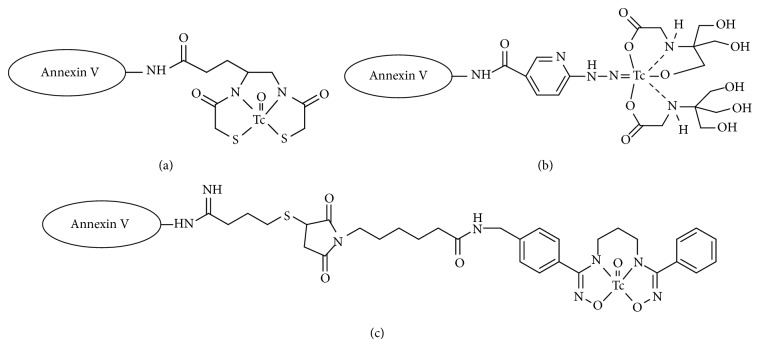
Structures of (a) ^99m^Tc-BTAP-annexin V, (b) ^99m^Tc-HYNIC-annexin V, and (c) ^99m^Tc-C_3_(BHam)_2_-annexin V.

^99m^Tc is an ideal radionuclide for scintigraphic imaging applications because of its excellent physical properties, low cost, and ready availability as a generator-produced nuclide. Since most polypeptides do not possess binding sites allowing them to form ^99m^Tc chelates with high* in vivo* stability, appropriate chelating molecules are incorporated into polypeptide molecules to prepare ^99m^Tc-labeled peptides for* in vivo *applications. Tetradentate ligands, such as N_3_S and N_2_S_2_ coordination molecules, form stable ^99m^Tc complexes with the [Tc=O]^3+^ core. In the field of apoptosis imaging, ^99m^Tc-4,5-bis(thioacetamido)pentanoyl(BTAP)-annexin V (Figure 1(a)) was developed as the first ^99m^Tc-labeled annexin V compound, which is a ^99m^Tc complex with N_2_S_2_ ligand-conjugated annexin V, after development of iodine-labeled annexin V. The first report of ^99m^Tc-BTAP-annexin V use was in thrombosis imaging and not for apoptosis imaging. In that paper, Stratton et al. reported that ^99m^Tc-BTAP-annexin V detected acute left atrial thrombi* in vivo* in swine because PS becomes exposed on the surface of activated platelets [6]. Narula et al. reported that ^99m^Tc-BTAP-annexin V was injected into cardiac allograft recipients. Some patients who showed at least moderate transplant rejection had positive myocardial uptake of radioactivity. This study showed the clinical feasibility of ^99m^Tc-labeled annexin V imaging for the noninvasive detection of apoptosis [7]. However, N_2_S_2_ ligands may require harsh conditions such as high pH or high temperature to prepare ^99m^Tc complexes with high radiochemical yields. Accordingly, ^99m^Tc labeling of annexin V with a N_2_S_2_ ligand was performed using a preformed-chelate approach. The conjugation between annexin V and ^99m^Tc complex must be performed after the complexation reaction of ^99m^Tc. This method requires multiple steps and purification, resulting in a 25%–30% overall radiochemical yield. For routine clinical use, ^99m^Tc labeling of annexin V, which can be prepared by an easier labeling operation, is required.

## 4. ^99m^Tc-HYNIC-Annexin V

Blankenberg et al. reported the use of ^99m^Tc-labeled annexin V, which has hydrazinonicotinamide (HYNIC) as a ligand for technetium, for apoptosis imaging [8]. HYNIC is one of the most attractive bifunctional chelating agents for the labeling of peptides and proteins with ^99m^Tc. It has been reported that HYNIC acts as a monodentate or bidentate ligand to form a mixed ligand ^99m^Tc complex in the presence of appropriate coligands [9]. Tricine has been frequently used as a coligand since it provides ^99m^Tc-HYNIC-labeled peptides and proteins with high radiochemical yields and high specific activities in a short reaction time at room temperature. ^99m^Tc-HYNIC-annexin V (Figure 1(b)) could be prepared in high radiochemical yield without any purification after a one-step reaction. In cultures of Jurkat T-cell lymphoblasts induced to undergo apoptosis, the uptake of ^99m^Tc-HYNIC-annexin V directly correlated with the percentage of cells labeled by FITC-labeled annexin V, which has an affinity for PS nearly identical to that of the native (unlabeled) protein, as determined by flow cytometry (*r* ^2^ = 0.922) [10]. In another study, the bioactivity of HYNIC-annexin V was verified by measuring its binding to erythrocyte membranes containing exposed PS. The affinity of HYNIC-annexin V was comparable with that of native annexin V. The IC_50_ values, which were determined using a competition assay, were 10.1 ± 2.0 nmol/L and 6.8 ± 0.7 nmol/L, respectively [11]. Moreover, many studies have demonstrated that ^99m^Tc-HYNIC-annexin V accumulates in apoptotic cells and can be used to visualize apoptosis in animal models [12–15]. ^99m^Tc-HYNIC-annexin V can be considered as a benchmark in the field of apoptosis imaging, as the easy labeling method has made this tracer the most extensively investigated and the best characterized apoptosis-detecting radioligand [16].

## 5. Clinical Studies of ^99m^Tc-Labeled Annexin V in Oncology

For evaluation of the response to cancer therapy, ^99m^Tc-HYNIC-annexin V imaging should be performed before and after the starting point of treatment. In a clinical study reported by Kartachova et al. in 2007, ^99m^Tc-HYNIC-annexin V imaging in 16 non-small-cell lung cancer patients was performed before and within 48 hours after the initiation of platinum-based chemotherapy [17]. All patients, displaying imaging data confirming markedly increased uptake of annexin V in tumor, showed complete or partial response. A significant correlation (*r* ^2^ = 0.86; *P* = 0.0001) was found between the changes of annexin V uptake in tumor and the treatment outcome.

In another clinical study reported by Rottey et al. in 2006, changes in relative ^99m^Tc-HYNIC-annexin V tumor uptake in patients undergoing chemotherapy at baseline and at 5–7 and 40–44 hours after treatment initiation were investigated [18]. The tumor response was evaluated by response evaluation criteria in solid tumors (RECIST) and related to observed changes in the ratios of tumor activity to background activity. Responders to chemotherapy could be separated from nonresponders with a 94% accuracy (16/17 patients) by use of the sequential ^99m^Tc-HYNIC-annexin V imaging and a 25% change threshold.

Although these studies indicated that ^99m^Tc-HYNIC-annexin V could be a good predictor of the response to cancer therapy, the numbers of patients in these studies were small. Furthermore, further larger studies are necessary to confirm the utility of ^99m^Tc-HYNIC-annexin V for the prediction of therapeutic effects in early stages after chemotherapy initiation.

## 6. Other ^99m^Tc-Labeled Annexin V Compounds

Tait et al. reported the use of ^99m^Tc-labeled annexin V with site-specific labeling in 2000 [11]. In this study, annexin V derivatives, containing N-terminal extensions of seven amino acids for complexation with technetium, were produced by expression in* E. coli* (NH_2_Ala-Cys-Gly-Gly-Gly-His-Met-annexin V, NH_2_Ala-Gly-Gly-Cys-Gly-His-Met-annexin V, and NH_2_Ala-Cys-Gly-Cys-Gly-His-Met-annexin V). One derivative is predicted to form N_2_S_2_ chelation site, and two are predicted to form N_3_S site. The bioactivities of the mutant proteins were verified by measuring binding activity to erythrocyte membranes with exposed PS. The mutant proteins also had the same degree of IC_50_ values (9.3 ± 0.4, 10.3 ± 2.5, and 10.0 ± 2.8 nmol/L), which were determined for unlabeled proteins by competition assay, as HYNIC-annexin V (10.1 ± 2.0 nmol/L). The radiochemical yields of the mutant proteins with ^99m^Tc were approximately 90%.

Tait et al. reported another annexin V mutant-labeled with a Tc(I)-carbonyl complex in 2002 [19]. They prepared annexin V mutants with N-terminal extensions of three or six histidine residues for labeling with a Tc(I)-tricarbonyl core. Annexin V-123, which has an N-terminal extension of six histidine residues, could be superior to annexin V-122, which has three histidine residues, because ^99m^Tc-labeled annexin V-123 had higher radiochemical yield and radiochemical purity and experimentally demonstrated bioactivity in binding to erythrocytes.

We recently developed a novel ^99m^Tc-labeled annexin V, ^99m^Tc-C_3_(BHam)_2_-annexin V (Figure 1(c)), using a bis(hydroxamamide) derivative [C_3_(BHam)_2_] as a bifunctional chelating agent to decrease uptake and retention in nontarget tissues compared with ^99m^Tc-HYNIC-annexin V [20]. In biodistribution experiments in normal mice, ^99m^Tc-C_3_(BHam)_2_-annexin V showed a much lower kidney accumulation of radioactivity than ^99m^Tc-HYNIC-annexin V. In the organs for metabolism, such as liver and kidney, radioactivity after the injection of ^99m^Tc-HYNIC-annexin V was residual for a long time. On the other hand, radioactivity after the injection of ^99m^Tc-C_3_(BHam)_2_-annexin V gradually decreased. In experiments using tumor bearing mice, ^99m^Tc-C_3_(BHam)_2_-annexin V showed significantly increased tumor uptake after 5-FU treatment. The accumulation of radioactivity in tumor correlated positively with the counts of TUNEL-positive cells.

## 7. ^18^F-Labeled Annexin V

Apoptosis imaging probes for positron emission tomography (PET) are required because PET imaging has high spatial resolution and great sensitivity, and ^18^F-labeled annexin V has been developed. In 2003, Zijlstra et al. reported the preparation of ^18^F-labeled annexin V using N-succinimidyl 4-[^18^F]fluorobenzoate ([^18^F]SFB) as a labeling reagent [21]. The decay-corrected radiochemical yield was in the range of 15–20%. The ^18^F-labeled annexin V highly bound to apoptotic Jurkat T-cells, compared with nonapoptotic control cells. In 2004, Toretsky et al. also reported ^18^F-labeled-annexin V via [^18^F]SFB [22]. The overall decay-corrected radiochemical yield of ^18^F-labeled annexin V from ^18^F^−^ was comparable (17.6% ± 5.6%). In addition, Murakami et al. also reported ^18^F-labeled annexin V by a similar procedure in the same year [23]. In that study, accumulation of ^18^F-labeled annexin V in the infarct area using a rat model of myocardial ischemia and reperfusion was comparable to that of ^99m^Tc-labeled-annexin V with site-specific labeling [11]. However, the complicated radiosynthesis procedure and the low radiochemical yield of ^18^F-labeling (especially, in this study, approximately 10%, decay-corrected form [^18^F]SFB) were weak points for clinical use.

## 8. ^68^Ga-Labeled Annexin V

The radionuclide ^68^Ga has great potential for clinical PET and could become an attractive alternative to ^18^F because of its radiophysical properties, particularly as a generator-produced nuclide with a half-life (*T*_1/2_) of 68 minutes [24]. It does not require an on-site cyclotron and can be eluted on demand. In principle, the long half-life of the parent nuclide ^68^Ge (*T*_1/2_ = 270.8 days) provides a generator with a long life span. Wangler et al. reported a ^68^Ga-labeling technique for proteins using a sulfhydryl-derivatized chelator, 2,2′-(7-(1-carboxy-4-(2-mercaptoethylamino)-4-oxobutyl)-1,4,7-triazonane-1,4-diyl)diacetic acid (NODA-GA-T) in 2011 [25]. In that study, ^68^Ga-labeled annexin V was prepared within a short time of only 15 minutes. In a PET study with an animal model, the ^68^Ga-labeled annexin V accumulated in the apoptotic area by the myocardial infarction.

In the same year, Bauwens et al. reported site-specific ^68^Ga-labeled annexin V compounds, ^68^Ga-Dotamaleimide-conjugated Cys2-annexin V and ^68^Ga-Dotamaleimide-conjugated Cys165-annexin V [26]. Cys2-annexin V and Cys165-annexin V are variants of annexin V containing a single available cysteine residue at respective positions 2 and 165. Total synthesis time was approximately 55 minutes withan end-of-synthesis yield of 25% (43% if decay-corrected) in both cases. Both compounds showed well-preserved PS binding capacity and high* in vitro* stability in buffer and in plasma. Tumor uptake of ^68^Ga-Cys2-annexin V and ^68^Ga-Cys165-annexin V was low but significantly increased after cyclophosphamide and radiation therapy in a tumor model.

## 9. Radiolabeled PS Targeted Peptides

Annexin V is best known in the field of apoptosis imaging as a carrier because it has high affinity for PS and that radiolabeled annexin V compounds have been extensively investigated and well characterized. However, the use of annexin V, a protein, imposes limitations, such as slow pharmacokinetics and potential immunogenicity. In contrast to protein-based radioligands like radiolabeled annexin V, for low-molecular-weight compounds, it is not difficult to modify chemical structures to improve their biodistribution. Accordingly, several studies have been performed in recent years to develop low-molecular-weight apoptosis imaging agents.

In 1995, Igarashi et al. reported a 14-amino acid synthetic peptide, FNFRLKAGQKIRFG (PSBP-0), derived from PS decarboxylase bound to PS effectively and specifically [27]. However, the binding affinity of PSBP-0 was not very high.

**Figure 2:**
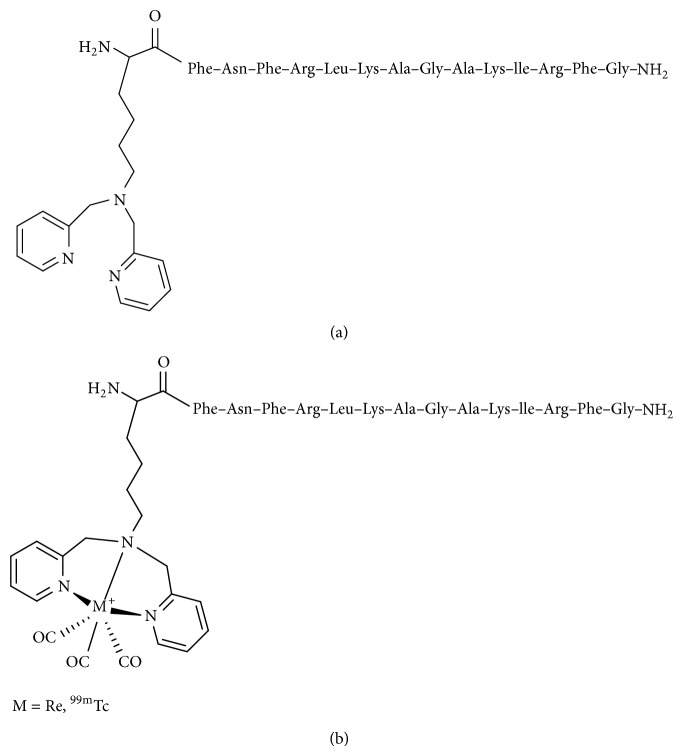
Structures of (a) SAAC-PSBP-6 and (b) SAAC(Re)-PSBP-6 and SAAC(^99m^Tc)-PSBP-6.

In 2011, Xiong et al. modified PSBP-0 to develop peptides with higher affinity for PS [28]. Each of the 14 residues of PSBP-0, except the endogenous alanine residue at position 8 (Ala^8^), was initially replaced with Ala to identify amino acid residues that contribute to PS affinity. The variant PSBP-6, in which Gln^6^ is replaced by Ala, showed the highest relative binding level and high stability. The binding levels of PSBP-6 were higher than those of PSBP-0. The remaining Ala-substituted peptides had lower affinity for PS than the parent PSBP-0 peptide. Next, Lys[di(2-pryidinemethyl)]-COOH, termed a single amino acid chelator (SAAC), was introduced at specific positions of PSBP-0 and PSBP-6 as a ligand for complexation with technetium. The peptide containing SAAC introduced at the N-terminus of PSBP-6 (SAAC-PSBP-6, Figure 2(a)) showed greater binding to PS than PSBP-6. Moreover, the rhenium complex, SAAC(Re)-PSBP-6 (Figure 2(b)), displayed a higher level of binding than SAAC-PSBP-6. In a biodistribution study, SAAC(^99m^Tc)-PSBP-6 (Figure 2(b)) showed significantly higher accumulation in B16/F10 melanoma treated with poly(l-glutamic acid)-paclitaxel than in untreated tumor (4.1 ± 0.6% ID/g versus 1.6 ± 0.3% ID/g).

In 2013, Song et al. compared SAAC(^99m^Tc)-PSBP-6 with [^18^F]FDG for detecting apoptosis induced by chemotherapy [29]. In B16/F10 melanoma and 38C13 lymphoma tumor models, the uptake of SAAC(^99m^Tc)-PSBP-6 significantly increased on the first day after treatment, whereas [^18^F]FDG uptake significantly decreased. The uptake of SAAC(^99m^Tc)-PSBP-6 negatively correlated with that of [^18^F]FDG (*r* = −0.79, *P* < 0.05). These results indicated that the SAAC(^99m^Tc)-PSBP-6 could be a useful probe to evaluate early therapeutic response after chemotherapy.

## 10. Bis(zinc(II)-dipicolylamine) Complex

**Figure 3:**
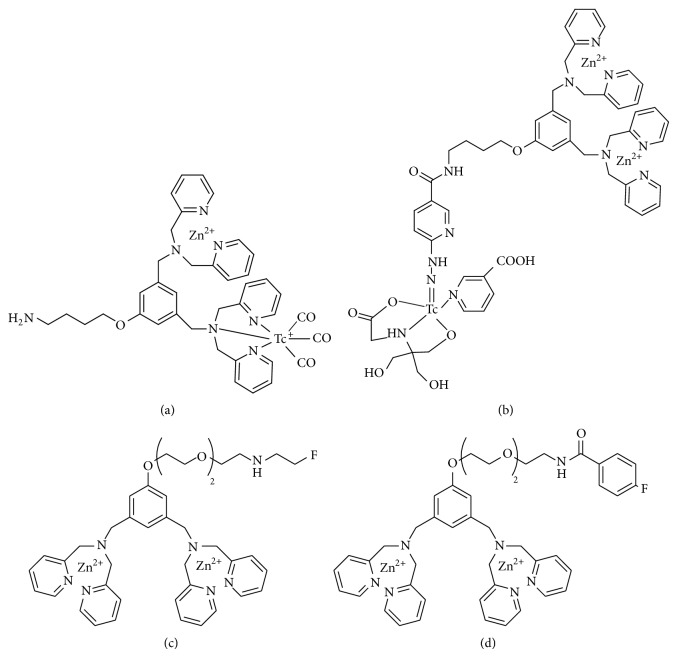
Structures of (a) ^99m^Tc-labeled Zn^2+^-DPA with ^99m^Tc-tricarbonyl core, (b) ^99m^Tc-labeled Zn^2+^-DPA with ^99m^Tc-HYNIC, (c) ^18^F-FEN-DPAZn2, and (d) ^18^F-FB-DPAZn2.

Binuclear Zn(II) complexes of 2,2′-dipicolylamine (Zn^2+^-DPA) are known as an effective binding motif for phosphate anion [30]. Because PS is an anionic phospholipid, Zn^2+^-DPA has a selective affinity for biomembranes enriched with PS and could have an apoptosis sensing function. In 2011, Wyffels et al. reported ^99m^Tc labeled Zn^2+^-DPA with a ^99m^Tc-tricarbonyl core and via ^99m^Tc-HYNIC (Figures 3(a) and 3(b)) [31]. Both compounds showed significantly higher uptake in livers of anti-Fas antibody-treated mice compared with that in livers of control mice. The increased liver uptake of ^99m^Tc-labeled Zn^2+^-DPA was comparable to that of ^99m^Tc-labeled annexin V, indicating the* in vivo* affinity of ^99m^Tc labeled Zn^2+^-DPA for hepatic apoptosis.

Recently, Wang et al. reported the ^18^F-labeled Zn^2+^-DPA compounds, 2-^18^F-fluoroethyl-bis(zinc(II)-dipicolylamine) (^18^F-FEN-DPAZn2) and 4-^18^F-fluoro-benzoyl-bis(zinc(II)-dipicolylamine) (^18^F-FB-DPAZn2) (Figures 3(c) and 3(d)), as apoptosis (cell death) imaging agents [32]. Decay-uncorrected radiochemical yields of the precursors of ^18^F-FEN-DPAZn2 and ^18^F-FB-DPAZn2, ^18^F-FEN-DPA2 and ^18^F-FB-DPA2, were 8.9% and 13%, respectively. ^18^F-FEN-DPAZn2 and ^18^F-FB-DPAZn2 complexes were prepared from ^18^F-FEN-DPA2 and ^18^F-FB-DPA2 with Zn(NO_3_)_2_. The authors described that the low radiochemical yield of ^18^F-FEN-DPA2 limited further animal experiments.* In vivo* PET imaging experiments using ^18^F-FDG, ^18^F-FB-DPA2, and ^18^F-FB-DPAZn2 were performed in Hepa1-6 hepatocellular carcinoma-bearing mice and adriamycin-treated tumor-bearing mice. ^18^F-FDG and ^18^F-FB-DPA2 showed no significant uptake change before and after chemotherapy. These results indicate that ^18^F-FDG could lack specificity to monitor anticancer therapy and that ^18^F-FB-DPA2 has no binding affinity to PS without zinc. In contrast, ^18^F-FB-DPAZn2 showed significantly higher accumulation in adriamycin-treated tumors compared with that in untreated ones. However, the background uptake of ^18^F-FB-DPAZn2 in liver and intestine was high. Optimization of the chemical structure is needed to improve its biodistribution to obtain images with higher signal/noise (*S*/*N*) ratio.

## 11. ^18^F -Labeled 5-Fluoropentyl-2-methyl-malonic Acid (^18^F -ML-10)

Several physiologically relevant processes, such as apoptosis, platelet activation, neurotransmitter release, and sperm capacitation, require dissipation of plasma membrane lipid asymmetry, a process known as scrambling. Apoptosis-related PS exposure on the cell surface is a classic feature of apoptotic cells and acts as an “eat me” signal allowing phagocytosis of postapoptotic bodies [33].

**Figure 4:**
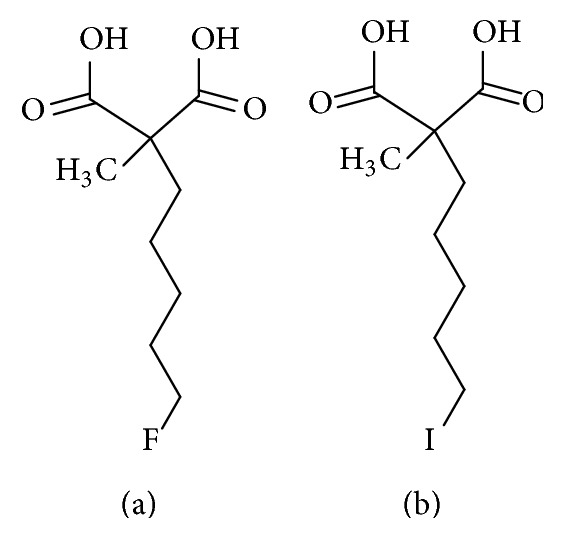
Structures of (a) ^18^F-ML-10 and (b) ^123^I-ML-10.

A family of small molecules, ApoSense, rationally designed to detect apoptosis-related complexes of cellular alterations, with consequent selective accumulation within apoptotic cells driven by the apoptotic scramblase activation, depolarization, and cellular acidification, have been reported as compounds capable of discriminating between vital and apoptotic cells [34]. In the ApoSense family, ^18^F-labeled 5-fluoropentyl-2-methyl-malonic acid (^18^F-ML-10, Figure 4(a)) was reported by Reshef et al. as a PET tracer of cell death in 2008 [35]. ^18^F-ML-10 showed fast blood clearance and little accumulation in normal tissues and rapid elimination via kidneys. Stability of ^18^F-ML-10 was very high and no metabolite was observed in blood and the brain. The absence of accumulation in bone indicated that defluorination of ^18^F-ML-10 did not occur. In an experimental acute cerebral stroke mouse model, ^18^F-ML-10 was retained in the stroke area but was cleared from intact brain areas. In 2011, a favorable dosimetry and safety profile of ^18^F-ML-10 were demonstrated in first human study [36]. Binding to apoptotic sites was also demonstrated.

The molecular design of ^18^F-ML-10 comprised the following steps: (1) attachment of an alkyl chain to render the molecule amphipathic for membrane interaction, (2) optimization of the alkyl-chain length, based on pharmacokinetic considerations, (3) addition of a methyl group at the *α*-position to prevent metabolism* in vivo*, and (4) attachment of a fluorine atom for PET imaging with ^18^F. To identify the mechanism of action, Cohen et al. synthesized and evaluated ^3^H-ML-10 [37]. The uptake of ^3^H-ML-10 in apoptotic cells occurred in parallel to annexin V binding. Approximately 10-fold higher accumulation of ^3^H-ML-10 was observed in anti-Fas antibody-treated apoptotic Jurkat cells compared with that in untreated control cells. ^3^H-ML-10 crossed the cell membrane at the stage of early apoptosis and was mainly localized in the cytoplasm (60%) and in the nucleus (30%). Interestingly, ^3^H-ML-10 accumulated only in apoptotic cells and not in either untreated control cells or necrotic cells induced by freeze-thaw injury.

Recently, ^123^I-labeled ML-10 analog as a single photon emission tomography (SPECT) tracer was reported. [^123^I]-2-(5-iodopentyl)-2-methylmalonic acid (^123^I-ML10, Figure 4(b)) was expected to show increased penetration in apoptotic cells because of its greater lipophilicity compared with ^18^F-ML-10 [38]. ^123^I-ML10 accumulated in apoptotic cells, but a clear uptake in the thyroid, resulting from* in vivo* deiodination, was observed because of its instability.

## 12. Caspase-3 Activity Imaging Agents

The caspases are a family of intracellular cysteine aspartate-specific proteases that play an important role in the initiation and execution of apoptosis [39]. Caspases are subdivided into three groups on the basis of homology and substrate specificity: (1) caspases involved in inflammation (caspases 1, 4, 5, and 13); (2) initiator caspases which are found at the top of the signaling cascade (caspases 6 and 8–10), and (3) effector caspases which are activated further downstream (caspases 2, 3, and 7) [40]. Among these caspases, caspase-3 is a frequently activated death protease, catalyzing the specific cleavage of many key cellular proteins [41]. Thus, caspase-3 should be an attractive biomarker for apoptosis.

**Figure 5:**
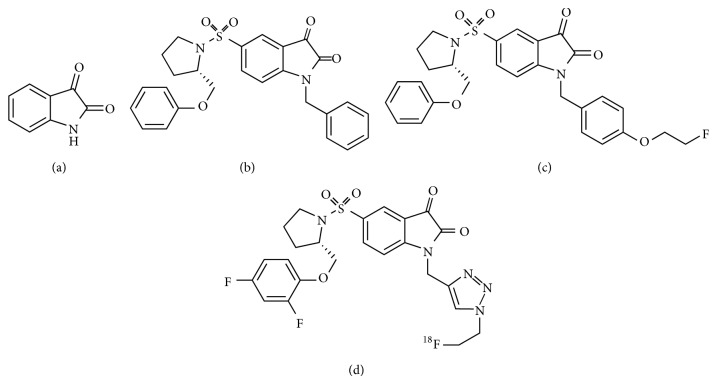
Structures of (a) Isatin, (b) (*S*)-1-benzyl-5-[1-[2-(phenoxymethyl)pyrrolidinyl]sulfonyl]isatin, (c) WC-II-89, and (d) [^18^F]ICMT-11.

In 2001, Lee et al. determined the activity and the selectivity of a series of isatin (Figure 5(a)) analogs for caspase-3 [42]. They found that the substituent at position 5 was important and demonstrated a correlation between the electron-withdrawing ability of the substituent at position 5 and the potency of caspase-3 inhibition. The 5-dialkylaminosulfonylisatins were identified as potent inhibitors of caspases 3 and 7. Within this series, (*S*)-1-benzyl-5-[1-[2-(phenoxymethyl)pyrrolidinyl]sulfonyl]isatin (Figure 5(b)) was the most promising and showed very high activity and selectivity for caspases 3 and 7.

In 2006, Zhou et al. synthesized WC-II-89 (Figure 5(c)), an isatin sulfonamide analog containing a fluorine atom that has a structure similar to that of compound 5B [43]. WC-II-89 was a highly potent inhibitor of caspases 3 and 7 and displayed high selectivity with respect to other caspases. [^18^F]WC-II-89 was prepared via a nucleophilic substitution of the corresponding mesylate precursor with [^18^F]fluoride ion in high radiochemical yield. In animal experiments, [^18^F]WC-II-89 showed higher uptake in the liver of a cycloheximide-treated rat, a model for chemically induced apoptosis, compared to the untreated control. Western blot analysis confirmed that the uptake was related to caspase-3 activation.

In 2008, Smith et al. reported the synthesis and characterization of the ^18^F-labeled isatin analog (*S*)-1-[[1-(2-fluoroethyl)-1*H*-[1,2,3]triazol-4-yl]methyl]-5-[2-(2,4-difluorophenoxy)methyl-pyrrolidine-1-sulfonyl]isatin ([^18^F]ICMT-11, Figure 5(d)). [^18^F]ICMT-11 possesses the characteristic of high metabolic stability with no indication of defluorination* in vivo*, reduced lipophilicity, and subnanomolar affinity for caspase-3 [44]. “Click labeling” provided [^18^F] ICMT-11 in 65% decay-corrected radiochemical yield from 2-[^18^F]fluoroethylazide.

In 2009, Nguyen et al. reported the results of a detailed* in vitro* study and an animal PET study of [^18^F]ICMT-11 [45].* In vitro* binding of [^18^F]ICMT-11 was increased in drug-treated cells and corresponded to higher cellular activity of caspases 3 and 7. In addition, [^18^F]ICMT-11 binding did not increase in caspase-3 deficient MCF-7 human breast cancer cells treated with 4-hydroperoxycyclophosphamide (4-HC), compared with a control. In the PET study, the [^18^F]ICMT-11 signal increased in up to twofold during the first 24 hours after treatment. Other papers have also recently indicated that [^18^F]ICMT-11 could be useful as a caspase-3 imaging apoptosis tracer [46, 47].

In 2013, Challapalli et al. reported the result of a PET study in healthy human volunteers that revealed the biodistribution and internal dosimetry profiles of [^18^F]ICMT-11 [48]. [^18^F]ICMT-11 was shown to be a safe PET tracer with a favorable radiation dosimetry profile for clinical use. Further clinical studies of [^18^F]ICMT-11 for apoptosis imaging are anticipated.

## 13. Radiolabeled Duramycin

**Figure 6:**
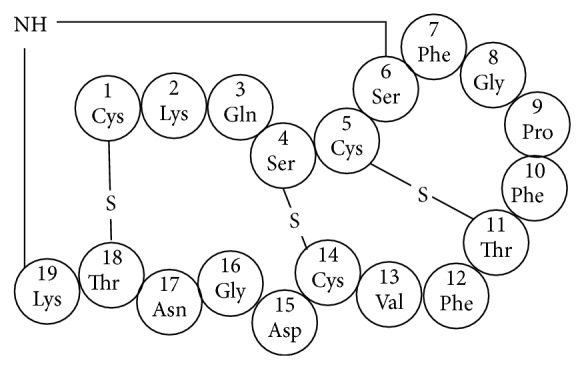
Structure of duramycin.

Phosphatidylethanolamine (PE) is the second most abundant phospholipid and accounts for approximately 20% of all phospholipids in mammalian cellular membranes [49]. Similar to PS, PE is a major component of the inner leaflet of the cell membrane and is rare on the surface of normal viable cells [50]. When apoptosis occurs, PE is exposed onto the cell surface [51]. Thus, PE could also be a target molecule for apoptosis imaging. As a potential molecular probe candidate for PE, duramycin (2 kDa), which is produced by* Streptoverticillium cinnamoneus*, is a tetracyclic peptide consisting of 19 amino acids (Figure 6) [52, 53] and binds the head group of PE with high affinity (*K*_*d*_ = 11 nM) at a molar ratio of 1 : 1 [54–56].

In 2008, Zhao et al. reported ^99m^Tc-labeled duramycin, which is a novel PE-binding molecular probe, using a HYNIC ligand with tricine and phosphine as coligands [57]. In a myocardial ischemia/reperfusion injury lesion using a rat model, ^99m^Tc-duramycin showed specific higher uptake in apoptotic cells compared with that in viable control cells. Intravenously injected ^99m^Tc-duramycin showed favorable pharmacokinetic and biodistribution profiles. It was rapidly cleared from the circulation via the renal system with a blood half-life of less than 4 minutes in rats. Hepatic and gastrointestinal uptake was very low. In addition, ^99m^Tc-duramycin was evaluated for ischemia/reperfusion injury in brain using the rat model of middle cerebral artery occlusion (MCAO) [58], oxidative lung injury [59], and damage in susceptible tissues after high-dose radiation exposure [60]. In all cases, ^99m^Tc-duramycin could detect apoptotic cells and was useful for the estimation of the degree of the symptoms.

Recently, Yao et al. reported ^18^F-labeled duramycin, [^18^F]FPDuramycin, as a novel tracer for PET imaging of cell death [61]. [^18^F]FPDuramycin was successful in visualizing tumor cell death after treatment with cyclophosphamide and cisplatin in tumor-bearing mice using PET. However, its pharmacokinetics was not favorable because [^18^F]FPDuramycin showed high accumulation in the liver and spleen, which would limit detection of cell death in the upper abdomen.

## 14. [^18^F]-*p*-Fluorobenzyl Triphenylphosphonium Cation (^18^F-FBnTP)

It is known that mitochondria play an important role in an intrinsic pathway of apoptosis. Disruption of the mitochondrial membrane potential (ΔΨ_m_) and mitochondrial outer membrane permeabilization, which leads to the release of intermembrane proteins including cytochrome c and others, occurs at an early stage of apoptosis [62, 63]. The collapse of ΔΨ_m_ occurs before the major morphology and biochemistry changes of apoptosis including nuclear DNA fragmentation and externalization of membranous PS [63]. Moreover, although the transient exposure of the PS allows measurement of the extent of the apoptosis during a limited time window, the loss of ΔΨ_m_ is an ongoing process not limited to a time window. Thus, monitoring ΔΨ_m_ could offer the information about the kinetics of the apoptotic process in the target tissue and could accordingly be an effective approach for detecting apoptosis at an early stage.

Phosphonium cations can pass through the lipid bilayer because they are sufficiently lipophilic and their positive charge is delocalized. In addition, because the membrane potential of mitochondria is the highest in cells, phosphonium cations could selectively accumulate in the innerior of the mitochondria [64]. Consequently, when apoptosis causes loss of ΔΨ_m_, phosphonium cations in cells would decrease.

**Figure 7:**
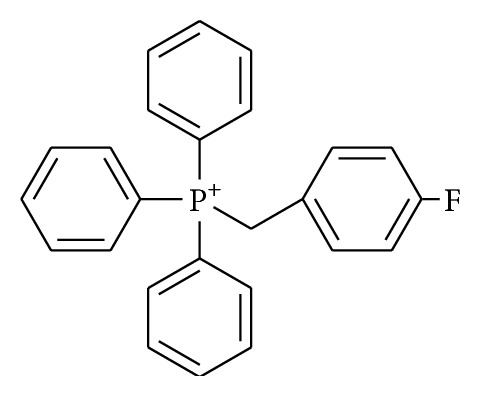
Structure of [^18^F]-*p*-fluorobenzyl triphenylphosphonium cation (^18^F-FBnTP).

In 2007, Madar et al. reported the ^18^F-labeled phosphonium cation, ^18^F-fluorobenzyl triphenylphosphonium cation (^18^F-FBnTP, Figure 7), as a PET tracer for noninvasive assessment of ΔΨ_m_ [65]. The performance of ^18^F-FBnTP for measuring changes in membrane potential was comparable to that of ^3^H-tetraphenylphosphonium (^3^H-TPP), which has been used for measurement of ΔΨ_m_ *in vitro*.* In vitro* experiments assessing the dependence of ^18^F-FBnTP uptake on membrane potential showed that about 80% of total uptake was ΔΨ_m_-dependent, about 10% was plasma membrane potential (ΔΨ_p_)-dependent, and remaining about 10% was independent of membrane potential, namely, nonspecific binding. Therefore, the change of radioactivity accumulation in the target tissue determined using ^18^F-FBnTP PET imaging could mainly reflect changes in ΔΨ_m_.

In 2009, Madar et al. reported* in vivo* study of ^18^F-FBnTP [66]. Tumor uptake under anticancer treatment was evaluated in orthotropic prostate tumor-bearing mice. At 48 hours after treatment with docetaxel, ^18^F-FBnTP and ^18^F-FDG were intravenously administered. At 60 minutes after injection, ^18^F-FBnTP uptake (percentage of dose per gram) in the prostate tumor of untreated mice was 1.84 ± 0.65 and that of treated mice was 0.90 ± 0.31. In contrast, ^18^F-FDG uptake levels in the prostate tumors of untreated mice and of treated mice were 1.31 ± 0.14 and 1.15 ± 0.54, respectively. Treatment with docetaxel resulted in a significant decline in tumor uptake of ^18^F-FBnTP (54.2%), but ^18^F-FDG showed no significant change in tumor uptake on treatment with docetaxel. Thus, the measurement of ΔΨ_m_ using ^18^F-FBnTP could be an effective strategy for the early detection of apoptosis.

## 15. Summary

In this review, we introduced a variety of apoptosis imaging probes. The most typical probes were radiolabeled agents that bind to PS, such as radiolabeled annexin V. Studies in model systems as well as clinical studies have demonstrated that the accumulation of PS-directed probes correlates with apoptotic cells after therapy. However, PS-directed probes accumulate not only as a result of apoptosis but also as a result of other forms of cell death. Both PS and PE are exposed onto the cell surface during apoptosis. PE-directed probes, such as radiolabeled duramycin, could also be apoptosis imaging agents using the same strategy used with PS-directed probes. Unlike PS- or PE-directed probes, caspase-3 imaging probes were expected to specifically detect apoptosis. Promising caspase-3 imaging probes have been developed, and their accumulation in apoptosis in animal models was shown to be higher than that in controls. However, the specificity of the imaging probe [^18^F]ICMT-11, which targets caspases 3 and 7, for the detection of apoptotic cells was not shown [45]. The small-molecule ^18^F-ML-10 (MW 206) is an apoptosis probe for PET that has simple structure derived from the ApoSense family. Although ^18^F-ML-10 binds at apoptotic sites, the absolute uptake of ^18^F-ML-10 in apoptotic tissue could be not enough. The probe ^18^F-FBnTP, which is used to assess ΔΨ_m_, can detect apoptosis because disruption of ΔΨ_m_ occurs at an early stage of apoptosis. The loss of ΔΨ_m_ is not limited to a specific time window, unlike the transient nature of PS exposure. A disadvantage of probes that assess ΔΨ_m_ is that their uptake may display a negative correlation with apoptosis.

Quantitative imaging of apoptosis (cell death) provides useful information for therapeutic effects on diseases before anatomical changes of the lesion site. Selection of the appropriate therapies on the basis of the imaging data from the patients may be possible. Because the apoptosis imaging can allow evaluating the therapeutic effects of drugs under development, this modality may help the screening of novel compounds. Some promising agents have been developed in recent years as we introduced some of them in the review, but their imaging quality is not always sufficient. We hope that novel apoptosis imaging agents, showing higher *S*/*N* ratios, will be developed in the near future.
